# Ultrasound vs. Fluoroscopy Guided Erector Spinae Plane Block in Postherpetic Neuralgia: A Prospective Study

**DOI:** 10.3390/jcm15114014

**Published:** 2026-05-22

**Authors:** Burcu Ozalp, Gunay Yolcu, Meltem Uyar, Can Eyigor

**Affiliations:** 1 Division of Pain Medicine, Department of Anesthesiology and Reanimation, Ege University, Izmir 35100, Turkey; meltemuyar@gmail.com (M.U.); can.eyigor@yahoo.com.tr (C.E.); 2 Department of Physical Medicine and Rehabilitation, Celal Bayar University, Manisa 45030, Turkey; dryolcugunay@gmail.com

**Keywords:** fluoroscopy, management, pain, neuralgia, postherpetic, ultrasound, interventional

## Abstract

**Background:** The erector spinae plane block (ESPB) is a commonly used interventional method in the treatment of post-herpetic neuralgia (PHN). ESPB can be performed under the guidance of ultrasound or fluoroscopy, and there are limited data in the literature regarding the effectiveness of ESPB using these two imaging modalities on pain. In this study, we aimed to compare the effectiveness of ESPB performed under the guidance of ultrasound and fluoroscopy on pain reduction in PHN. **Methods:** Patients between the ages of 18 and 90 years with thoracal pain related to PHN and scheduled to undergo the ESPB were enrolled in the prospective observational study. Patients were divided into two groups: those who underwent a single-shot fluoroscopy-guided (FS) or ultrasound-guided (USG) ESPB. The Numeric Rating Scale-NRS was used to assess pain intensity, and the DN-4 questionnaire was used to assess the presence of neuropathic pain (NP). **Results:** In the final analysis, data from a total of 48 patients, 24 from each group, were evaluated. No statistically significant difference was found between the two groups in terms of demographic data and initial clinical features. While a significant decrease in NRS scores was seen in both groups at the first and third months, no significant difference was seen between the two groups. There was no significant change in the number of patients with NP in either group compared to baseline. **Conclusions:** Both ultrasonography and fluoroscopy-guided ESPB appear to be effective in reducing pain in PHN. Further studies are needed for the efficacy of ESPB on NP.

## 1. Introduction

Post-herpetic neuralgia (PHN) is defined as dermatomal pain persisting for at least 90 days after the initial onset of the herpes zoster rash [1,2]. Pathogenetically, PHN results from the reactivation of the varicella-zoster virus, which leads to inflammatory damage of peripheral nerves, followed by peripheral and central sensitization. Epidemiologically, the prevalence of PHN increases significantly with age and immunosuppression, with severe acute pain and ophthalmic involvement serving as well-defined risk factors [3,4].

The management of PHN necessitates a multidisciplinary approach due to its treatment-resistant nature. This comprehensive framework incorporates pharmacological options such as gabapentinoids, tricyclic antidepressants, and topical lidocaine patches, alongside non-pharmacological interventions including transcutaneous electrical nerve stimulation (TENS), psychological counseling, and physical therapy to address the multifaceted nature of chronic pain [4,5].

Interventional pain management emerges as a pivotal option for cases unresponsive to these conservative treatments. Various established techniques, including epidural steroid injections, paravertebral blocks, sympathetic nerve blocks, and neuromodulation, have been extensively employed in clinical practice. However, the search for safer and more easily applicable alternatives has led to the emergence of fascial plane blocks. Among these, the erector spinae plane block (ESPB) has gained significant attention [5,6,7,8,9,10]. In the ESPB procedure, an injectate is administered into the fascial plane deep to the erector spinae muscle and superficial to the tips of the vertebral transverse processes, targeting the dorsal and ventral rami of the spinal nerves [11,12]. While early evidence for ESPB in PHN is promising, it often relies on case series, and its comparative efficacy across different imaging modalities remains under-researched.

As with all interventional procedures, image guidance is mandatory to ensure precision and minimize serious complications such as pneumothorax or neuraxial spread [13]. Ultrasonography is currently considered the gold standard for fascial plane blocks due to its real-time soft tissue visualization. However, in specific clinical scenarios—such as in patients with high body mass index where ultrasound waves may have poor penetration, or in cases of distorted anatomy—fluoroscopy provides an essential anatomical landmark-based alternative. Despite these potential clinical differences in applicability and operational efficiency, there is a lack of prospective literature directly comparing the clinical efficacy and pain reduction outcomes of these two imaging modalities. Therefore, the present study aimed to compare the effectiveness of ultrasound-guided versus fluoroscopy-guided ESPB in patients with PHN.

In this context, the erector spinae plane block (ESPB) is utilized as a regional technique where an injectate containing local anesthetic, with or without steroids, is administered into the fascial plane deep to the erector spinae muscle and superficial to the tips of the vertebral transverse processes [6]. The analgesic mechanism is primarily attributed to the local spread of the injectate to the dorsal and ventral rami of the spinal nerves [8].

Interventional pain management serves as a pivotal option for PHN cases unresponsive to conservative treatment. While various methods such as paravertebral blocks, epidural steroid injections, and neuromodulation have been employed, several studies indicate that ESPB may be an applicable alternative in managing zoster-related pain [9,10,11,12,13,14]. However, these reports often rely on case series or observational data. As with all interventional pain procedures, image guidance is recommended to enhance both safety and accuracy [15]. While ESPB is typically performed under ultrasonography, fluoroscopy-guided applications have also been reported; however, there is a lack of prospective literature directly comparing the clinical efficacy of these two imaging modalities.

Therefore, the novelty of this study lies in evaluating fluoroscopy as an alternative imaging modality and directly comparing its clinical effectiveness with the current standard of ultrasound guidance. The primary objective of this study was to evaluate the reduction in pain intensity at the one-month follow-up (primary endpoint), as assessed by the Numerical Rating Scale (NRS), in patients with postherpetic neuralgia (PHN) receiving either ultrasound-guided or fluoroscopy-guided ESPB. Additionally, the study aimed to compare these two imaging modalities regarding their impact on neuropathic pain scores and analgesic consumption over a three-month period.

## 2. Methods

### 2.1. Study Design and Population

This prospective observational study was conducted in two tertiary center hospitals between May 2024 and January 2025. Patients aged between 18 and 90 years who had been experiencing postherpetic neuralgia-related complaints in the thoracal region for a minimum of three months and who had not responded adequately to medical treatment were included in the study. The diagnosis of PHN was made on the basis of anamnesis and physical examination findings. Patients with a documented history of zoster infection and complaints such as pain, burning, tingling, and electric shock-like sensations in specific dermatomal areas for a minimum duration of three months following the rash were included in the study.

The study excluded individuals with a history of previous interventional procedures for PHN, patients who had interventional pain management or surgery for another etiology in the thoracal region within the last 3 months, patients with disk herniation and spinal stenosis that could cause comparable findings in the thoracal region and lead to diagnostic confusion, those with systemic infection or skin infection findings in the procedure area, pregnant and breastfeeding women, those with a mental disorder that would affect the evaluation, and those with an allergic history to the drugs to be administered.

This study was approved by the local ethics committee (date: 2 May 2024, number: 1580). Verbal and written informed consent were obtained from all patients participating in the study.

### 2.2. Procedures

Patients were allocated to either the fluoroscopy (FS) or ultrasonography (USG) group based on the availability of imaging equipment at the time of the procedure, provided that both techniques were considered clinically appropriate. No randomization was performed. The allocation process was conducted by an independent researcher who was not involved in the interventional procedure or outcome assessment. This study was designed as a prospective, observational analysis of routine clinical practice. The choice of imaging modality was independent of the research protocol and was dictated by standard institutional care for post-herpetic neuralgia (PHN) management. The first group underwent ultrasonography-guided ESPB (USG group), while the second group underwent fluoroscopy-guided ESPB (FS group). The target level at which ESPB was applied was determined on the basis of the patient’s anamnesis and physical examination findings. All patients received a single-session interventional procedure. No repeat blocks were performed during the follow-up period. All ESPB procedures were performed by pain medicine specialists, each having at least 10 years of clinical experience in both ultrasound and fluoroscopy-guided interventional techniques. This ensured that the technical execution of the blocks was standardized and performed at an expert level across both imaging groups, minimizing potential bias related to the learning curve or operator experience. Before the procedure, the patients were conveyed to the operating room, where they were positioned in ventral decubitus (prone position) on the operating table and monitored. The injection site was sterilized and a covering applied. The procedures were performed utilizing a 22-gauge, 90 mm spinal needle.

**Figure 1:**
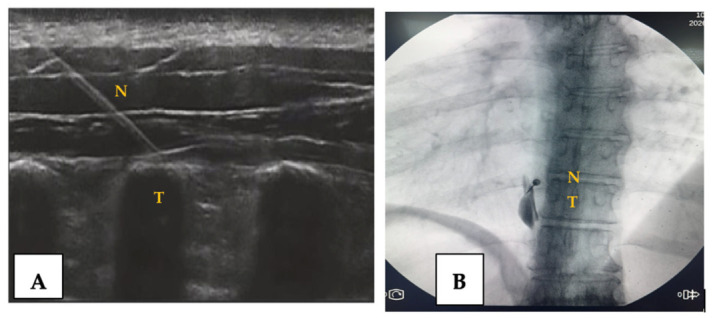
(**A**) The ultrasound-guided ESPB technique and needle placement. (**B**) The fluoroscopy-guided ESPB technique and needle placement. (N: needle T: transverse process).

In the USG group, unilateral ESPB was performed at the affected side using an ultrasonography machine (Chison SonoAir 70 ultrasound system (CHISON Medical Technologies Co., Ltd., Wuxi, Jiangsu, China). The laminae were enumerated from the sacrum to the target level of injection. The high-frequency probe was placed longitudinally in the midline over the vertebral spine of the targeted level. The probe was then slid laterally until the transverse process and paraspinal muscles were identified. The needle was then introduced from the cephalic aspect of the probe, targeting the transverse process. Negative aspiration was done to exclude intravascular placement of the tip of the needle. Then, a total of 20 milliliters of a previously prepared mixture consisting of 40 milligrams of triamcinolone acetonide, 10 milliliters of bupivacaine, and 9 milliliters of 0.9% physiological serum was injected in the plane between the transverse process and the fascia of the erector spinae muscle with confirmation of the spread of the injectate by ultrasonography. The ultrasound-guided ESPB procedure and needle placement are illustrated in Figure 1A.

In the FS group, the target level was determined by first counting up from the sacrum and confirming with the help of the ribs. The anteroposterior image of the targeted thoracal region was viewed under fluoroscopy (OEC One mobile C-arm fluoroscopy system (GE Healthcare, Chicago, IL, USA)) with the spinous processes in the midline. The needle was advanced under sequential fluoroscopy imaging with the tunnel vision technique, targeting the lateral end of the transverse process on the relevant side. Upon reaching the targeted area, a contrast agent was utilized to ascertain the absence of intravascular spread, thereby confirming the attainment of the desired fascial spread pattern. Subsequently, an identical mixture was injected into the area of interest, as was performed in the USG group. The fluoroscopy-guided ESPB procedure and needle placement are illustrated in Figure 1B.

Following the completion of all pertinent procedures, patients were subjected to a two-hour monitoring period for the identification of any potential adverse effects. Any such effects that were observed were documented.

### 2.3. Outcome Measures

The clinical and demographic data of all patients were recorded, including age, height, weight, duration of symptoms, medications used, any accompanying diseases, and dermatomal levels of complaints. The Numeric Rating Scale was employed to evaluate the intensity of pain, while the DN-4 (Douleur Neuropathique-4) questionnaire was utilized to ascertain the presence of neuropathic pain.

Numeric rating scale (NRS): The NRS is a frequently used method for measuring pain severity and monitoring progress. The scale comprises 11 points, ranging from 0 for no pain to 10 for the most severe possible pain. Patients are asked to rate their pain between 0 and 10.

Douleur Neuropathique-4 (DN-4) Questionnaire: The DN-4 questionnaire is a simple and rapid-to-administer diagnostic tool employed for the identification and assessment of neuropathic pain. Neuropathic pain-related 10 items were grouped under 4 questions. 7 items are derived from the patient’s description of their pain, while 3 items are based on the sensory examination conducted by the clinician [15]. A score of 4 or more suggests the presence of neuropathic pain [15].

The data obtained from the NRS and DN-4 assessments were recorded at the pre-intervention period and at 24 h, 1st and 3rd months post-intervention follow-up. All evaluations were conducted by a separate clinician, who was not involved in the procedure. The primary endpoint of this study was the reduction in pain intensity at the 1-month follow-up, as assessed by the Numerical Rating Scale (NRS). The secondary endpoints were defined as changes in NRS scores at the 3-month post-procedure mark, the presence of neuropathic pain evaluated via the Douleur Neuropathique 4 (DN-4) questionnaire, and the monitoring of daily analgesic requirements and medication dosages throughout the entire 3-month follow-up period.

### 2.4. Statistical Analyses

Statistical analysis was performed using SPSS version 23.0 (IBM Corp., Armonk, NY). The sample size was determined using G*Power software (version 3.1.9.4). Based on a predicted difference of 2 points in the NRS score (14) between groups at the 1st month—representing the minimally clinically important difference (MCID)—with an alpha (α) level of 0.05 and a power (1 − β) of 0.80, the power analysis indicated that a minimum of 44 patients (22 per group) were required for the study.

The normality of data distribution was evaluated using the Shapiro–Wilk test. For intergroup comparisons of continuous variables (age, BMI, NRS scores, and medication dosages), the Independent Samples *t*-test was employed for normally distributed data. To account for the longitudinal nature of the repeated measurements within subjects, a Linear Mixed-Effects Model (LMM) framework was utilized. Individual patients were entered into the model as random effects (random intercept), while the imaging modality (Group), follow-up intervals (Time), and the Group × Time interaction were specified as fixed effects. This framework was applied to analyze the changes in NRS scores and daily analgesic medication dosages over time. To control for Type I error inflation due to multiple comparisons, the Bonferroni correction was applied to all post hoc and paired-samples tests.

Categorical data, including gender and the presence of neuropathic pain according to DN-4 scores, were analyzed using the Chi-Square test or Fisher’s Exact test. To evaluate the magnitude of clinical effect, Cohen’s d coefficients and 95% confidence intervals were calculated. For all analyses, a *p*-value < 0.05 was considered statistically significant.

## 3. Results

**Figure 2:**
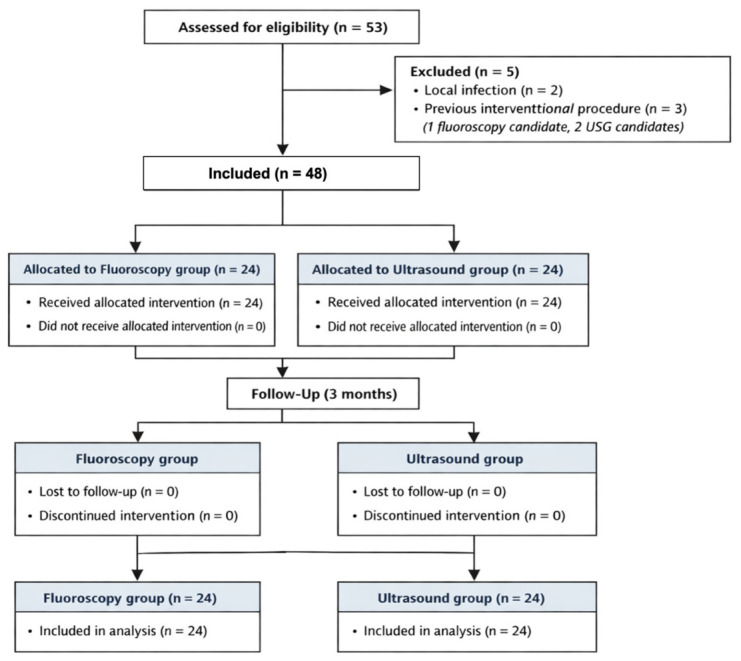
Flow Diagram of patient enrollment, allocation, follow-up, and analysis (*n*: number of patients).

In the final analysis, data from a total of 48 patients, 24 from each group, were evaluated. No statistically significant difference was found between the two groups in terms of demographic data and initial clinical features (Table 1). The thoracal level and the side on which the procedure was performed are also documented in Table 1, in accordance with the dermatomal levels at which the complaints manifested. The study flow is shown in Figure 2.

The analysis of time-dependent change in NRS revealed a statistically significant reduction in the scores for all follow-up periods in both groups, compared to the initial measurement. The Linear Mixed-Effects Model (LMM) analysis for longitudinal NRS data demonstrated a highly significant main effect for time (*p* < 0.001). Compared to baseline, a substantial and statistically significant reduction in pain scores was observed across both groups at 24 h (β = −8.70, z = −29.78, *p* < 0.001), 1 month (β = −6.90, z = −23.62, *p* < 0.001), and 3 months (β = −4.50, z = −15.40, *p* < 0.001). The Group × Time interaction effects were statistically non-significant at all follow-up intervals: 24 h (*p* = 1.000), 1 month (β = 0.20, z = 0.48, *p* = 0.628), and 3 months (β = −0.21, z = −0.52, *p* = 0.606), indicating that the trajectory of pain relief did not differ significantly between the two imaging modalities at any follow-up point. While no significant difference was observed between groups, the effect sizes (Cohen’s d) for all comparisons were small, further supporting the comparable efficacy of both imaging modalities (Table 2). A comparison of the number of patients with neuropathic pain according to DN-4 revealed no significant difference from baseline to all follow-up periods in both groups (Table 3).

Confidence interval analysis confirmed that all between-group comparisons for pregabalin and gabapentin crossed zero, indicating no meaningful difference between FS and USG groups. For tramadol, although effect sizes at the 1st and 3rd months suggested a small-to-moderate advantage for the USG group, confidence intervals remained wide and crossed zero. In contrast, within-group analyses demonstrated large and clinically significant reductions in tramadol consumption in both groups, with consistently larger effect sizes in the USG group. Changes in drug doses over time are given in Table 4.

**Figure 3:**
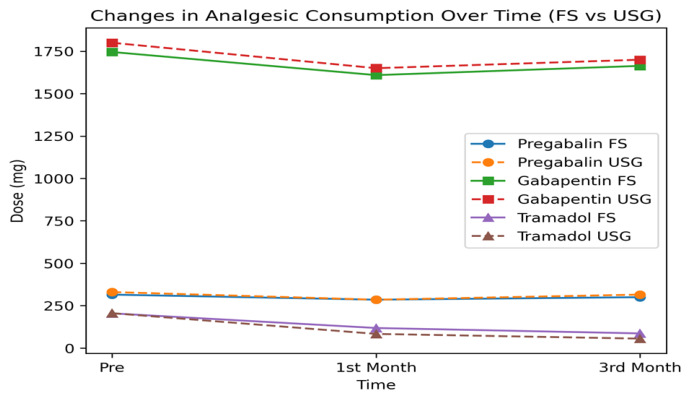
Comparison of pregabalin, gabapentin, and tramadol consumption between FS and USG groups at baseline, 1st month, and 3rd month.

There were no statistically significant differences between the FS and USG groups in terms of pregabalin, gabapentin, or tramadol consumption at baseline, 1st month, or 3rd month (*p* > 0.05 for all comparisons). Although not statistically significant, tramadol reduction tended to be greater in the USG group. The data are presented graphically in Figure 3.

Parallel LMM analyses executed for daily analgesic consumption confirmed significant main effects for time (*p* < 0.05) but failed to show any significant Group × Time interaction effects (*p* > 0.05), indicating a balanced reduction in medication requirements between the groups.

In the course of the ESPB procedure, four patients (three in the USG group, one in the FS group) exhibited transient hypotension, which was successfully managed with basic supportive measures. Three patients (one in the USG group, two in the FS group) experienced a transient increase in pain following the procedure. No significant adverse effects or complications were observed in any of the patients.

## 4. Discussion and Conclusions

The findings of the present study suggest that ESPB resulted in significant pain reduction with both ultrasound and fluoroscopy-guided techniques. Although no statistically significant difference was observed between the two groups, both techniques demonstrated significant improvement compared to baseline. However, no significant effect was observed on the number of patients with neuropathic pain according to DN-4. To the best of our knowledge, this is the first prospective study in the literature comparing the effectiveness of USG- and fluoroscopy-guided ESPB in patients with PHN.

The clinical efficacy of the erector spinae plane block (ESPB) in PHN is primarily attributed to the local anesthetic’s blockade of nociceptors and nerve endings within the targeted fascial plane [16]. When the administered local anesthetic can permeate the vascular system and exert a systemic effect, further research is required to ascertain the extent to which this phenomenon contributes to the observed outcomes [16]. Based on the view that the most prominent effects are seen in the local environment, confirmation of the localization where the injectate is applied is important. Ultrasonography is a valuable diagnostic and therapeutic tool that can be used to evaluate muscle tissue, fascia structure, and other tissues in real-time. During injection, this technique allows for direct visualization of the needle’s position and its relationship with muscle and fascia, facilitating precise targeting and minimizing potential vascular damage. Although the spread can be observed with the aid of a contrast agent in fluoroscopy, the disadvantage is that the area of injection application cannot be fully visualized and soft tissues cannot be evaluated.

Although ESPB is most commonly performed under ultrasonographic guidance in both the literature and daily practice, there is data indicating that fluoroscopy guidance is also valid. In a case report, ESPB was successfully applied under fluoroscopic guidance in severe chest pain due to rib fracture, although it is etiologically different from PHN [17]. Although radiation exposure is the most important disadvantage, fluoroscopy may be preferred in cases where ultrasonography is not available or when there is no clinician with sufficient experience in the use of ultrasonography. The use of fluoroscopy may also be considered in cases where ultrasonography may be technically inadequate, such as in obese patients. While no statistically significant difference was found in pain scores between fluoroscopy and ultrasonographic guidance in our sample, this does not formally imply equivalence. The balanced physical characteristics between groups minimize BMI-related anatomical depth as a confounder in this comparison, representing a methodological strength. However, our results suggest that fluoroscopy may be considered as a feasible imaging option in situations where ultrasonography is not available or not technically applicable. Further research is necessary to ascertain its technical and clinical utility.

A multitude of factors may exert an influence on clinical efficacy that extends beyond the imaging guidance that will be utilized. One of the most significant issues in the domain of ESP application pertains to the determination of the optimal volume of injectate to be administered. Cadaver studies have demonstrated that as the volume of injectate administered increases, cephalocaudal spread of local anesthetic occurs at a greater level [18]. In one of the most recent cadaveric studies, it was demonstrated that with an injectate volume of 30–40 mL, the spread occurred at 4–7 paravertebral levels, and with a volume of 60–80 mL, it occurred at 12–13 paravertebral levels [18]. Further studies are needed to determine the optimal dose that will produce the most clinical efficacy and the least side effect profile.

The optimal timing of ESPB intervention remains a critical factor in the management of zoster-associated pain. While the efficacy of the block in the chronic phase (PHN) is well-documented, emerging evidence suggests that interventions performed during the acute phase may halve the incidence of PHN development [19]. However, the precise impact of early-stage blocks on pain chronicity and neuropathic progression remains to be fully elucidated. Determining the definitive therapeutic window—both pre- and post-PHN development—warrants further prospective investigation.

In our study, a significant reduction in NRS scores was observed up to the third month following a single-shot ESPB. While some literature suggests that the most profound therapeutic effects of ESPB are achieved during the acute phase of herpes zoster [20], our results indicate that even in the chronic phase, a single intervention containing steroids may provide prolonged relief. This sustained effect might be attributed to the potent anti-inflammatory properties of triamcinolone acetonide and the potential of the block to interrupt the cycle of peripheral and central sensitization, though the underlying mechanism in chronic PHN requires further investigation.

A notable finding in our study was the significant reduction in NRS scores without a corresponding change in the number of patients categorized with neuropathic pain via the DN-4 questionnaire. This discrepancy requires careful interpretation. The DN-4 is a qualitative screening tool where a score (greater than or equal to 4) indicates the presence of neuropathic pain characteristics [21]. This suggests that while ESPB reduced pain intensity, neuropathic pain characteristics, as assessed by DN-4, may have persisted in this study. This indicates that while the ‘volume’ of the pain was lowered, the neuropathic components—likely due to established central sensitization in chronic PHN—were not fully reversed by a single intervention. This also highlights a potential limitation of using binary screening tools like DN-4 to measure treatment response in chronic neuropathic states.

Furthermore, the transition from severe (NRS 9) to moderate (NRS 4) pain in a chronic, refractory condition like PHN represents a significant clinical success. According to IMMPACT recommendations, a ≥50% reduction in pain intensity is a ‘substantial improvement’ [22]. Therefore, the persistence of neuropathic symptoms despite lower pain intensity reflects the complexity of established neural damage rather than a failure of the intervention. This study demonstrates that ESPB serves as a key component of a multimodal regimen, providing meaningful relief in a difficult-to-treat patient population.

This study has several limitations. First, the absence of a placebo or sham-controlled group limits causal interpretation of the findings. However, as this study reflects real-world clinical practice, a placebo arm was not feasible. Nevertheless, the consistent reduction in pain scores suggests a clinically meaningful benefit. Future randomized controlled trials are needed. Furthermore, since this study was designed as an observational analysis of clinical practice, the lack of a pre-defined non-inferiority margin means our findings demonstrate comparable utility rather than statistical equivalence. Larger, controlled non-inferiority studies are required for formal validation. Moreover, although analgesic consumption was recorded and found to be balanced between groups at baseline, it was not included as a covariate in the statistical model to adjust for potential confounding. This lack of statistical adjustment remains a limitation in isolating the independent analgesic effect of the block from systemic medications.

As mentioned above, the presence of neuropathic pain was assessed using the questionnaire-based DN-4 scale, and other critical domains—such as quality of life—were not evaluated. Additionally, the follow-up period was limited to three months; thus, the long-term effects of ESPB were not investigated. Beyond these limitations, the present study is significant as the first to prospectively compare the effectiveness of USG- and fluoroscopy-guided ESPB in PHN. Although procedure time and patient satisfaction were not designated as primary endpoints, future research should evaluate these parameters, considering the real-time imaging advantages of ultrasound versus the definitive anatomical precision of fluoroscopy.

## Figures and Tables

**Table 1:** Demographical data, initial clinical features of the patients and intervention characteristics.

	Fluoroscopy (FS) *n* = 24		Ultrasonography (USG) *n* = 24		*p* Value
Age (years)	60.6 (6.8)		62.1 (6.2)		0.24
Gender	F:13	M:11	F:14	M:10	
Weight (kg)	65.6 (8.9)		67.4 (7.8)		0.16
Height (cm)	157.4 (9.8)		154.9 (8.2)		0.23
Symptom duration (months)	8.2 (2.4)		8.6 (1.8)		0.18
Pre-NRS	9.3 (0.3)		9.4 (0.2)		0.86
Drugs					
Paracetamol	3		2		0.64
Paracetamol + codeine	7		5		0.51
NSAID	2		1		0.55
Tramadol	11		9		0.57
Paracetamol + tramadol	8		10		0.57
Gabapentinoids	21		22		1.00
Duloxetine	3		2		0.64
Intervention characteristics					
Side (R/L)	R: 14	L:10	R:12	L:12	
Level					
T2	2		1		
T3	1		1		
T4	3		4		
T5	1		-		
T6	5		4		
T7	1		1		
T8	6		7		
T10	4		5		
T12	1		1		

Demographical data, initial clinical features of the patients and intervention characteristics (R: right, L: left). *n*: number of patients; SD: standard deviation. Values are presented as Mean (SD).

**Table 2:** NRS scores in FS and USG groups with between-group comparisons, intragroup changes, Cohen’s d and 95% CI.

NRS	FS Group Mean (SD)	USG Group Mean (SD)	*p* Value (Between)	Cohen’s d (FS vs. USG)	95% CI
Pre-NRS (I)	9.3 (0.3)	9.4 (0.2)	0.86	−0.05	−0.62 to 0.52
NRS-24 h (II)	0.6 (0.1)	0.7 (0.1)	0.94	−0.02	−0.59 to 0.55
NRS-1 m (III)	2.4 (0.8)	2.7 (1.1)	0.24	−0.31	−0.88 to 0.26
NRS-3 m (IV)	4.8 (2.4)	4.6 (2.3)	0.56	0.08	−0.47 to 0.63
Intragroup time-dependent comparisons	FS Group *p*-value	USG Group *p*-value			
I and II	<0.001	<0.001			
I and III	<0.001	<0.001			
I and IV	0.01	<0.001			

Comparison of the intergroup and time-dependent intragroup changes in NRS scores (FS: fluoroscopy, USG: ultrasound, 24 h: 24th hour, 1 m: 1st month, 3 m: 3rd month). *n:* number of patients; SD: standard deviation. Values are presented as Mean (SD).

**Table 3:** DN-4 scores in FS and USG groups with between-group comparisons, intragroup changes, Cohen’s d and 95% CI.

DN-4	FS Group	USG Group	*p* Value	Cohen’s d (FS vs. USG)	95% CI
Pre-DN4 (I)	24	24	1	0.00	−0.58 to 0.58
DN4-24 h (II)	19	18	0.96	0.12	−0.46 to 0.70
DN4-1 m (III)	21	21	1	0.00	−0.58 to 0.58
DN4-3 m (IV)	22	20	0.84	0.18	−0.40 to 0.76
Intragroup time-dependent comparisons	FS Group *p*-value	USG Group *p*-value			
I and II	0.56	0.43			
I and III	0.67	0.67			
I and IV	0.88	0.54			

Comparison of the intergroup and time-dependent intragroup changes in the number of patients with neuropathic pain according to the DN-4 scores (FS: fluoroscopy, USG: ultrasound, 24 h: 24th hour, 1 m: 1st month, 3 m: 3rd month).

**Table 4:** Change in total dosages of daily use drugs.

Group FS (mg) Mean (SD)	Pre-Intervention (I)	1st Month (II)	3rd Month (III)	I&II	I&III	Cohen’s d (I–II) (95% CI)	Cohen’s d (I–III) (95% CI)
Pregabalin	315 (131.34)	285 (131.34)	300 (122.47)	0.16	0.317	−0.23 (−0.79 to 0.33)	−0.12 (−0.68 to 0.44)
Gabapentin	1745.45 (480.34)	1609.09 (450.45)	1663.64 (508.47)	0.083	0.157	−0.29 (−0.85 to 0.27)	−0.17 (−0.73 to 0.39)
Tramadol	204.55 (87.90)	118.18 (90.20)	86.36 (97.70)	0.002	0.001	−0.97 (−1.58 to −0.36)	−1.45 (−2.14 to −0.76)
**Group USG (mg) Mean (SD)**	**Pre-Intervention (I)**	**1st Month (II)**	**3rd Month (III)**	**I&II**	**I&III**	**Cohen’s d (I–II) (95% CI)**	**Cohen’s d (I–III) (95% CI)**
Pregabalin	330 (94.87)	285 (131.34)	315 (110.68)	0.083	0.317	−0.39 (−0.95 to 0.17)	−0.14 (−0.70 to 0.42)
Gabapentin	1800 (557.60)	1650 (503.62)	1700 (484.30)	0.180	0.317	−0.28 (−0.84 to 0.28)	−0.18 (−0.74 to 0.38)
Tramadol	205.56 (76.83)	83.33 (82.92)	55.56 (72.65)	<0.001	<0.001	−1.53 (−2.23 to −0.83)	−1.88 (−2.64 to −1.12)

Change in total dosages of daily use drugs (FS: fluoroscopy, USG: ultrasound, SD: standard deviation). *n:* number of patients; SD: standard deviation. Values are presented as Mean (SD).

## Data Availability

The data are not publicly available due to patient privacy and ethical restrictions. The statistical data underlying the findings of this study can be made available by the corresponding author upon reasonable request.
